# Pain Phenotypes in Endometriosis: A Population‐Based Study Using Latent Class Analysis

**DOI:** 10.1111/1471-0528.18021

**Published:** 2024-12-03

**Authors:** Fleur Serge Kanti, Valérie Allard, Andrée‐Ann Métivier, Madeleine Lemyre, Kristina Arendas, Sarah Maheux‐Lacroix

**Affiliations:** ^1^ Centre Hospitalier Universitaire de Québec – Université Laval Quebec City Quebec Canada

**Keywords:** endometriosis, latent class analysis, pain phenotype, pelvic pain, quality of life, subgrouping

## Abstract

**Objective:**

To identify pain phenotypes in patients with endometriosis and investigate their associations with demographics, clinical characteristics, comorbidities and pain‐related quality of life (QoL).

**Design:**

Cross‐sectional, single‐centre, population‐based study.

**Setting:**

Referral university centre in Quebec City, Canada.

**Population:**

Patients diagnosed with endometriosis were enrolled consecutively between January 2020 and April 2024.

**Methods:**

Latent class analysis was used to identify pain phenotypes. A three‐step approach of latent class analysis, involving logistic regression models, was applied to assess the associations between pain phenotypes and demographics, clinical characteristics, comorbidities and pain‐related QoL.

**Main Outcome Measures:**

Pain phenotypes; demographic, clinical and comorbidity predictors of phenotype membership; association between QoL and pain phenotypes.

**Results:**

A total of 352 patients were included. Two pain phenotypes were identified with distinct clinical presentations: one (54% of the participants) with more severe and frequent pain symptoms and poorer QoL and the other (46% of the participants) with mild and less frequent pain symptoms. The high pain phenotype was associated with previous treatment failure, painkiller use, familial history of endometriosis, low annual family income and comorbidities, including painful bladder, fibromyalgia, migraines, lower back pain, irritable bowel syndrome, anxiety and depression or mood disorders. The presence of endometrioma was associated with the low pain phenotype. Phenotype membership was associated with distinct QoL profiles (*p* < 0.001). The mean QoL score was higher in the high pain phenotype (59; 95% CI, 56–62) than in the low pain phenotype (33; 95% CI, 29–37).

**Conclusion:**

Patients with endometriosis can be categorised into two distinct phenotypes that correlate with QoL and patient characteristics. Validation in other populations is necessary and could aid the development of specialised or personalised interventions.

## 1 Introduction

Endometriosis is a complex and highly heterogeneous condition associated with a variety of chronic pain symptoms, including dysmenorrhea, dyspareunia, dysuria, dyschezia and chronic pelvic pain [1]. Diverse clinical features and chronic and complex pain syndromes in patients with endometriosis challenge the identification and targeting of pain drivers and optimization of patient management. Current medical and surgical therapies are not curative and focus on symptom control. Responses to hormonal therapy can range from complete remission of symptoms to the progression of lesions [2, 3], while lesions may progress, regress or even remain unchanged after surgery [4, 5]. Our understanding of the variations between patient presentation and treatment response remains limited. Current classification systems (revised American Society for Reproductive Medicine [6], ENZIAN [7] and American Association of Gynecologic Laparoscopists [8] classifications) are excellent for describing the extent of endometriosis lesions in the pelvis but poorly correlate with the severity of pain symptoms [9, 10].

Endometriosis leads to systemic inflammation and alters gene expression in the brain, which can cause pain sensitization and mood disorders [11]. A better understanding of the pathogenesis of endometriosis is needed, including identifying precise macrolevel or molecular ‘subphenotypes’. Endometriosis has been traditionally classified based on anatomy [12, 13] and recently on the stromal‐immune microenvironment and gene expression [14, 15, 16, 17]. An innovative and emerging statistical approach to pain research involves pain phenotyping [18, 19, 20]. The Initiative on Methods, Measurement and Pain Assessment in Clinical Trials group defined the phenotype as an ensemble of observable characteristics displayed by an organism with a special focus on patient‐self‐reported characteristics (e.g., psychosocial functioning), patient‐reported symptoms (e.g., sleep disruption) and verbal or behavioural responses to standardised psychophysical tests of pain sensitization [20]. Individuals with the same phenotype could share their clinical presentation, risk factors, prognosis and response to therapy. Furthermore, medical societies have emphasised the need to investigate whether meaningful pain phenotypes can be derived from the data of people with endometriosis and whether they can be linked to outcomes of interest to them [21].

In the present study, we aimed to identify pain phenotypes in patients with endometriosis and investigate their associations with demographics, clinical characteristics, comorbidities and pain‐related quality of life.

## 2 Methods

### 2.1 Study Design and Population

Data for this population‐based and cross‐sectional study were obtained between January 2020 and April 2024 from an ongoing longitudinal study conducted at the *Centre Hospitalier Universitaire de Québec‐Université Laval*, a referral university centre for endometriosis in Quebec City, Canada. Consecutive patients diagnosed with endometriosis or presenting with pelvic pain were invited to participate in the study by a member of the medical team (attending, student or nurse) at the time of consultation at the outpatient gynaecology clinic or by a research professional screening the patient list for eligibility within 2 weeks prior to the visit. Patients were sent a link by email to complete the consent form (ethical approval reference number from the local research ethics committee: 2020‐4972) and study questionnaires. The study population was limited to individuals aged 18 to 50 years with a diagnosis of endometriosis. The diagnoses were established through clinical suspicion, which included symptomatology and physical examination; imaging modalities; or histological confirmation [1, 13, 22]. Patients who reported no pain over the past 12 months and those with pain related to pregnancy, childbirth, sports or other injuries were excluded from the study. Patients diagnosed with cancer were also excluded from this study.

### 2.2 Data Collection and Variables

Variables were collected using the Endometriosis Patient Questionnaire [23] (self‐administered by participants), the Physical Examination Standards in Endometriosis and Pain Form [24] (completed by the medical team) and the Standard Surgical Form [25] (completed by the surgeons) from the Endometriosis Phenome and Biobanking Harmonisation Project of the World Endometriosis Research Foundation, and the Endometriosis Health Profile (EHP‐30) questionnaire [26] (self‐administered by participants).

#### 2.2.1 Latent Class Analysis Indicators (Input Variables)

To derive latent pain phenotypes, we used the following input variables:

1. Frequency of pelvic pain: monthly, weekly and daily.
2. Severity within the last 3 months of superficial dyspareunia, deep dyspareunia, dysmenorrhea, dyschezia and acyclic pelvic pain. The intensity of each pain symptom was measured using an 11‐point numeric rating scale [27] and the severity of each symptom was then assessed as a three‐level ordered categorical variable: none or mild pain (0–3), moderate pain (4–6) and severe pain (7–10) [28].
3. Pain quality was assessed using 14 descriptors from the 20‐item Pain Quality Assessment Scale (PQAS) [29] that were grouped into three pain quality factors: paroxysmal pain (PQAS descriptors: shooting, sharp, electric and hot), superficial pain (itchy, cold, numb, sensitive and tingling) and deep pain (aching, heavy, dull, cramping and throbbing) [30]. Participants rated each descriptor of pain quality as experienced in the previous 3 months on a scale of 0 (no pain or no ‘sensation/item’) to 10 (the most ‘descriptor’ pain sensation imaginable). Pain quality factors were classified as ‘severe’ if a minimum of one of their predefined descriptors received a rating of 7 or higher, and ‘up to moderate’ otherwise.

#### 2.2.2 Demographic, Clinical and Comorbidity Variables (Candidate Predictors of or Factors Associated With the Phenotypes)

Demographic variables included age, self‐identified ethnic or cultural origin, employment status, marital status, education level and annual family income. Clinical characteristics included age at menarche, nulliparous status, first‐degree relative history of endometriosis or chronic pelvic pain, body mass index, use of hormonal treatment within the last 3 months, use of painkillers within the last 3 months, failure (persistence of pain or intolerance) of previous treatment (combined hormonal contraceptives, progestins, agonists/antagonists of gonadotropin‐releasing hormone, neuromodulators, infiltrations, psychotherapy or physiotherapy), diagnostic method of endometriosis (histology, imaging and clinical impression) and type of endometriosis lesions (ovarian, superficial and deep endometriosis).

Fourteen comorbidities associated with endometriosis‐related pelvic pain were examined. These were reported by participants as having been experienced at some point in their medical history or diagnosed by a doctor as a cause of their general pelvic or lower abdominal pain. These are presented as four principal categories [12, 31]:

1. Gynaecologic or genitourinary conditions: ovarian cysts other than endometriomas, polycystic ovarian syndrome, uterine fibroids, adenomyosis, infertility and painful bladder or interstitial cystitis.
2. Rheumatologic and neurological conditions: fibromyalgia, chronic fatigue syndrome, migraine and low back pain.
3. Gastrointestinal conditions: irritable bowel syndrome and inflammatory bowel disease as Crohn's disease or ulcerative colitis.
4. Psychological conditions: anxiety and depression or mood disorders requiring medication or therapy.

#### 2.2.3 Pain‐Related Quality of Life (Outcome of Interest Across the Phenotypes)

The pain subscale (first 11 items) of the EHP‐30, ranging from 0 (best quality of life) to 100 (worst quality of life), was used to assess pain‐related quality of life over the past 4 weeks [26].

### 2.3 Data Statistical Analyses

**FIGURE 1:**
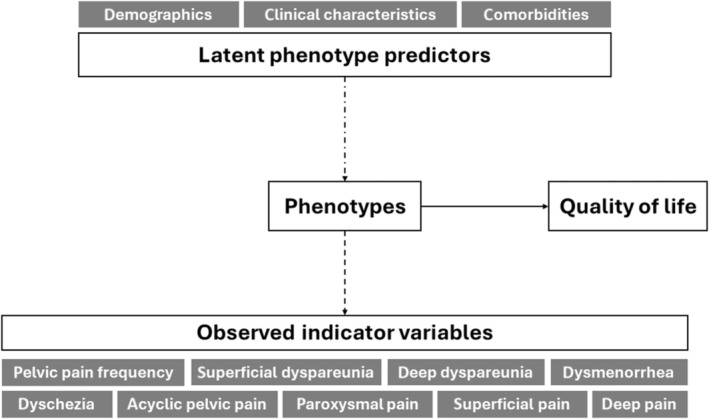
Diagram of the measurement and auxiliary models. The dashed black arrow represents the ‘measurement model’ (latent class or phenotype model), a model that defines the latent categorical variable (pain phenotypes) by referring to the set of observed or measured indicators (latent class analysis input variables). The dotted black arrow represents the ‘auxiliary model’, which uses a set of observed variables (demographics, clinical characteristics and comorbidities) as predictors of the latent categorical variable (pain phenotypes). The solid black arrow represents the ‘auxiliary model’, which uses the latent categorical variable (pain phenotypes) as a predictor of an observed variable called the distal outcome (here, quality of life). The frequency of pelvic pain was categorised as monthly (one or more days per month but not weekly), weekly (one or more days per week but not daily) and daily. The symptoms of pain were superficial, deep dyspareunia (pain that occurred on superficial, deep penetration of the vagina during intercourse), dysmenorrhea (pain that occurred during menses), dyschezia (painful bowel movements) and acyclic pelvic pain (pelvic/lower abdominal pain not related to intercourse, menstrual cramps or bowel movements and not caused by surgery, pregnancy or other injuries and infections). Severity for each pain symptom was assessed as a three‐level ordered categorical variable (none or mild pain (0–3), moderate pain (4–6) and severe pain (7–10)) based on an 11‐point numeric rating scale (the endpoints of the scale being 0 and 10 indicating ‘no pain’ and ‘worst possible pain’ respectively) reflecting the participant's level of pain in the past 3 months. Fourteen (14) descriptors from the 20‐item Pain Quality Assessment Scale (PQAS) were grouped into the three pain quality factors: Paroxysmal pain (PQAS descriptors: Shooting, sharp, electric and hot), superficial pain (itchy, cold, numb, sensitive and tingling) and deep pain (aching, heavy, dull, cramping and throbbing). Participants rated each descriptor of pain quality as experienced in the previous 3 months on a scale of 0 (no pain or no ‘sensation/item’) to 10 (the most ‘descriptor;’ pain sensation imaginable). Pain quality factors were categorised as ‘severe’ if at least one of their predefined descriptors was rated as 7 or higher, and ‘up to moderate’ otherwise. Demographic variables were age, self‐identified ethnic or cultural origin, employment status, marital status, education level and annual family income. Clinical characteristics were age at menarche, nulliparous status, first‐degree relative history of endometriosis or chronic pelvic pain, body mass index, use of hormonal treatment within the last 3 months, use of painkillers within the last 3 months, failures (persistence of pain or intolerance) of previous treatment (combined hormonal contraceptives, progestins, agonists/antagonists of gonadotropin‐releasing hormone, neuromodulators, infiltrations, psychotherapy or physiotherapy), diagnosis method of endometriosis (histology, imaging and clinical impression) and type of endometriosis lesions (ovarian, superficial and deep endometriosis). Comorbidities included gynaecologic or genitourinary conditions (ovarian cysts other than endometriosis, polycystic ovarian syndrome, uterine fibroids, adenomyosis, infertility and painful bladder or interstitial cystitis), rheumatologic and neurological conditions (fibromyalgia, chronic fatigue syndrome, migraine and low back pain), gastrointestinal conditions (irritable bowel syndrome and inflammatory bowel disease, such as Crohn's disease or ulcerative colitis) and psychological conditions (depression or mood disorders requiring medication or therapy and anxiety disorders requiring medication or therapy). Outcome (pain‐related quality of life) was assessed using the pain subscale of the validated 30‐item Endometriosis Health Profile Questionnaire. The recall period lasted for the last 4 weeks.

Data management, descriptive analysis and data visualisation were conducted in R software (version 4.4.1) [32]. Categorical variables are reported as frequencies (percentages) and continuous variables as means ± standard deviations (with median, range and interquartile range, where appropriate). Latent class and association analyses were performed using Mplus (version 8.11) [33] and a diagram of the measurement and auxiliary models is presented in Figure 1 (see Methods S1 for more details).

#### 2.3.1 Latent Class Analysis

We conducted a latent class analysis with up to 10 classes to identify pain phenotypes. The one‐class model was utilised as a baseline with subsequent increments in the number of classes until the optimal class solution model was achieved [34, 35]. Smaller Bayesian information criterion values indicate better model fit. The bootstrap likelihood ratio test was used to compare k‐class models to k−1‐class models (a significant *p*‐value favouring the k‐class model) [34, 36]. The certainty of classification was assessed using entropy. Utilising posterior probabilities, we assigned each participant to the class with the highest probability of belonging. This means that individuals with hypothetical posterior probabilities of 5%, 10%, 40%, 30% and 15% belonging to classes 1, 2, 3, 4 and 5, respectively, were assigned to class 3.

#### 2.3.2 Covariates and Outcome Association Analyses

The relationships between the identified latent classes and the participants' demographic and clinical characteristics as well as comorbidities were examined using logistic regression models in a bias‐adjusted three‐step procedure of latent class analysis with covariates. This methodology estimates the measurement model, assigns subjects to latent classes based on their calculated probabilities of class membership and correlates class assignments with an external variable while accounting for potential uncertainty in the classification process. The three‐stage procedure was also used to test whether the latent classes were associated with quality of life [33, 37, 38]. Odds ratios (ORs) were calculated and reported with 95% confidence intervals (CIs). Significant differences were assessed by examining CIs.

## 3 Results

### 3.1 Sample Characteristics

In total, 352 participants met the inclusion and exclusion criteria. The participants' characteristics are presented in Table 1. The mean age of the study population was 36 ± 7 years old. The participants identified themselves mostly as North Americans (83%). The participants predominantly reported having university education (44%), current employment (80%) and a family annual income of $60 000 or above (54%). The majority of participants were in committed relationships (82%), including dating, marriage or common‐law partnerships, and were nulliparous (56%). The average time since the onset of symptoms was 12 ± 9 years. More than one‐third of the participants (35%) had a first‐degree relative with a history of endometriosis or chronic pelvic pain. In the last 3 months, 45% and 66% used hormonal therapy and pain killers respectively. More than half of the population (52%) was overweight or obese (mean body mass index: 27 ± 7). Previous treatment failure was noted in 58% of the patients. The median number of comorbidities per patient was 4 (range: 0–12) with the most prevalent comorbidities being lower back pain (83%), uterine fibroids (72%), migraines (45%), anxiety disorders (44%), depression or mood disorders (35%) and adenomyosis (34%). Most patients (69%) had endometriosis confirmed by histology (*N* = 135) or imaging (*N* = 106). Diagnosed lesions (not mutually exclusive) were deep (90%) and superficial (23%) endometriosis and endometrioma (29%).

**TABLE 1:** Sample characteristics stratified by pain phenotypes identified.

Variables	Pain phenotypes^a^		Total (*N* = 352)
	Low (class 1) *n* = 163 (46.3%)	High (class 2) *n* = 189 (53.7%)	
Age (years)			
18–30	32 (19.6%)	61 (32.3%)	93 (26.4%)
31–40	82 (50.3%)	71 (37.6%)	153 (43.5%)
41–50	49 (30.1%)	57 (30.2%)	106 (30.1%)
Cultural/ethnic origin: North American^b^	135 (82.8%)	158 (83.6%)	293 (83.2%)
Education level			
Elementary/high school^c^	34 (21.0%)	48 (25.4%)	82 (23.4%)
Collegial	47 (29.0%)	67 (35.4%)	114 (32.5%)
University	81 (50.0%)	74 (39.2%)	155 (44.2%)
Working status			
Working currently	143 (87.7%)	139 (73.5%)	282 (80.1%)
Not currently working not due to pelvic pain	19 (11.7%)	23 (12.2%)	42 (11.9%)
Not currently working due to pelvic pain	1 (0.6%)	27 (14.3%)	28 (8.0%)
Annual family income			
≤ $19 999/preferred not to answer/NA	23 (14.1%)	44 (23.3%)	67 (19.0%)
$20 000–$59 999	44 (27.0%)	52 (27.5%)	96 (27.3%)
$60 000–$99 999	41 (25.2%)	46 (24.3%)	87 (24.7%)
≥ $100 000	55 (33.7%)	47 (24.9%)	102 (29.0%)
Marital status: dating/married/common law^d^	135 (82.8%)	154 (81.5%)	289 (82.1%)
Nulliparous	88 (54.0%)	109 (57.7%)	197 (56.0%)
Age at menarche: ≤ 13 years^e^	123 (75.5%)	141 (74.6%)	264 (75.0%)
Years since onset of symptoms	12.2 ± 9.7 (0–39)	12.4 ± 9.2 (0–32)	12.3 ± 9.4 (0–39)
First relative history^f^	45 (27.6%)	77 (40.7%)	122 (34.7%)
Use of hormones in the last 3 months	72 (44.2%)	85 (45.0%)	157 (44.6%)
Use of pain killers in the last 3 months	83 (50.9%)	148 (78.3%)	231 (65.6%)
Body mass index (kg/m^2^)			
Underweight (< 18.49)	2 (1.2%)	4 (2.1%)	6 (1.7%)
Normal weight (18.5–24.99)	75 (46.0%)	87 (46.0%)	162 (46.0%)
Overweight (25–29.99)	39 (23.9%)	47 (24.9%)	86 (24.4%)
Obese (≥ 30)	47 (28.8%)	51 (27.0%)	98 (27.8%)
Previous treatment failures^g^	80 (49.1%)	123 (65.1%)	203 (57.7%)
Diagnosis methods of endometriosis			
Histology	64 (39.3%)	71 (37.6%)	135 (38.4%)
Imaging	50 (30.7%)	56 (29.6%)	106 (30.1%)
Clinical impression	49 (30.1%)	62 (32.8%)	111 (31.5%)
Type of endometriosis lesions^h^			
Endometrioma	56 (34.4%)	45 (23.8%)	101 (28.7%)
Superficial endometriosis	36 (22.1%)	45 (23.8%)	81 (23.0%)
Deep endometriosis	145 (89.0%)	171 (90.5%)	316 (89.8%)
Number of comorbidities per patient	3.5 ± 2.1 (0–11)	5.0 ± 2.5 (0–12)	4.3 ± 2.4 (0–12)
Comorbidities^h^			
Ovarian cysts other than endometrioma	45 (27.6%)	56 (29.6%)	101 (28.7%)
Polycystic ovarian syndrome	16 (9.8%)	27 (14.3%)	43 (12.2%)
Uterine fibroids	44 (71.0%)	52 (73.2%)	96 (72.2%)
Adenomyosis	20 (31.7%)	28 (35.4%)	48 (33.8%)
Infertility	25 (15.3%)	19 (10.1%)	44 (12.5%)
Painful bladder/interstitial cystitis	27 (16.6%)	53 (28.0%)	80 (22.7%)
Fibromyalgia	6 (3.7%)	21 (11.1%)	27 (7.7%)
Chronic fatigue syndrome	10 (6.1%)	19 (10.1%)	29 (8.2%)
Migraines	61 (37.4%)	97 (51.3%)	158 (44.9%)
Low back pain	123 (75.5%)	168 (88.9%)	291 (82.7%)
Irritable bowel syndrome	28 (17.2%)	74 (39.2%)	102 (29.0%)
Inflammatory bowel disease^i^	1 (0.6%)	6 (3.2%)	7 (2.0%)
Depression or mood disorders^j^	46 (28.2%)	76 (40.2%)	122 (34.7%)
Anxiety disorders^j^	60 (36.8%)	95 (50.3%)	155 (44.0%)

Abbreviations: *n*, frequency per group; *N*, sample size; NA, not available.

^a^ Values are given as frequency (percentage) or mean ± standard deviation (range).

^b^ Versus Autochthon‐Inuit, Central and South America, European, African, Asian, mixed and others.

^c^ Or preferred not to answer.

^d^ Versus single, separated, divorced and widowed.

^e^ Versus > 13 years or were not sure.

^f^ History of endometriosis or chronic pelvic pain.

^g^ Persistence of pain or intolerance.

^h^ Diagnoses were not mutually exclusive.

^i^ Crohn's disease or ulcerative colitis.

^j^ Requiring medication or therapy.

### 3.2 Latent Phenotypes

The optimal model with diagonal elements in the average latent posterior probability matrix greater than 0.90 was the one with two classes of phenotypes (see Tables S1 and S2 for more details on different models tested). The high pain phenotype (class 2) presented higher probabilities for severe intensity and high frequency of all pain symptoms and qualities than the low pain phenotype (class 1) (Figure S1). Additionally, the mean or median scores for all pain symptoms were higher in the high‐pain group than in the low‐pain phenotype group (Table S3). The mean ranged from 3.2 ± 3.3 (for superficial dyspareunia) to 6.7 ± 3.5 (for dysmenorrhea) for the high pain phenotype, while it ranged from 1.9 ± 2.7 (for superficial dyspareunia) to 4.0 ± 3.2 (for deep dyspareunia) for the low pain phenotype. The median ranged from 2 (for superficial dyspareunia) to 8 (for dysmenorrhea) for the high pain phenotype, while it ranged from 0 (for superficial dyspareunia) to 4 (for deep dyspareunia or acyclic pelvic pain) for the low pain phenotype.

### 3.3 Descriptive Characteristics of Individuals in the High and Low Pain Phenotypes

The high pain phenotype represented approximately half of the sample (189 participants, 54%). Compared with the low pain phenotype, individuals with the high pain phenotype had a lower mean age and were more likely to be North Americans, unemployed and nulliparous. Additionally, they were less likely to have a college degree. The use of hormones or painkillers in the past 3 months was also more common in this group, as was previous treatment failure. Finally, individuals with a high pain phenotype had a greater number of comorbidities and a higher prevalence of all comorbidities, except infertility (Table 1).

### 3.4 Predictors of Phenotype Membership

**FIGURE 2:**
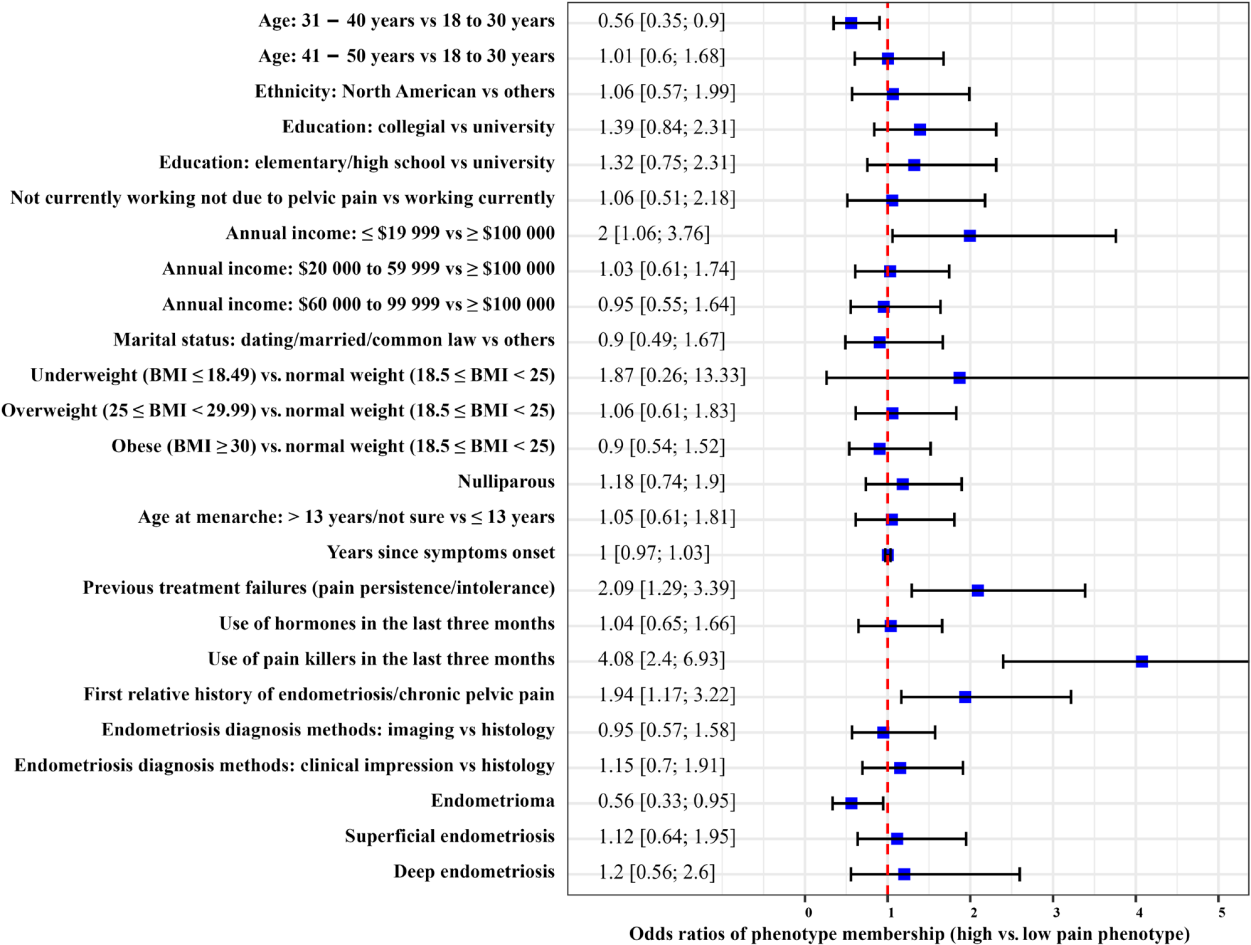
Unadjusted associations of phenotypes with demographic and clinical characteristics. BMI, Body Mass Index. The plot shows the results of the ‘auxiliary model’, which uses demographic and clinical characteristics as predictors of the latent categorical variable (phenotypes) in logistic regression models. The resulting odds ratios (ORs) were the odds of being a member of the high pain phenotype (relative to the low pain phenotype) in the category of a given variable (demographic and clinical characteristics) compared with the odds of membership in the indicated reference category of that variable (the reference category of binary variables is assumed to be the absence of the indicated category). For the continuous variable years since symptom onset, the OR is for each 1‐unit increase in the number of years since symptom onset. The ORs are indicated by the blue squares (point estimates), with their 95% confidence intervals delimited by the black horizontal solid lines. The red vertical dashed line represents the null value (1) of the OR and helps to identify an OR that is significantly different from 1 if its confidence interval does not include 1, that is, if the red vertical dashed line does not intersect the confidence interval line (corresponding to a *p*‐value < 0.05).

**FIGURE 3:**
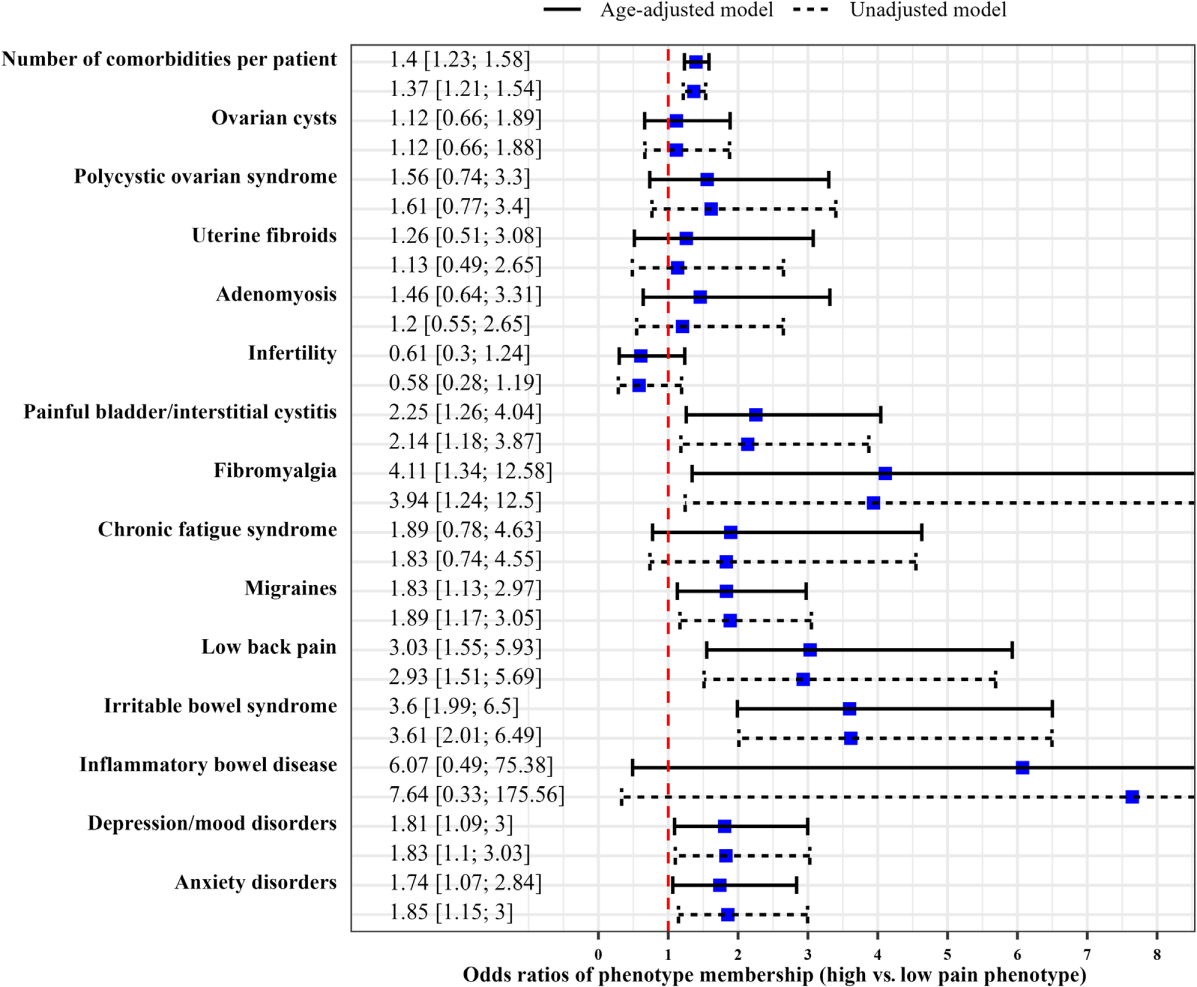
Unadjusted and age‐adjusted associations of phenotypes with comorbidities. The plot shows the results of the ‘auxiliary model’, which uses comorbidities as predictors of the latent categorical variable (phenotypes) in logistic regression models. The resulting odds ratios (ORs) were the odds of being a member of the high pain phenotype (relative to the low pain phenotype) in the presence of a given comorbidity compared to the odds of that membership in the absence of that comorbidity. For the continuous variable, the number of comorbidities per patient, the odds ratio (OR) was calculated for each unit increase in the number of comorbidities per patient. The ORs are indicated by the blue squares (point estimates), with their 95% confidence intervals delimited by the black horizontal solid and dashed lines (age‐adjusted and unadjusted models respectively). The red vertical dashed line represents the null value (1) of the OR and helps to identify an OR significantly different from 1 if its confidence interval does not include 1, that is, if the red vertical dashed line does not intersect the confidence interval line (corresponding to a *p*‐value < 0.05).

Participants aged 31–40 years compared to 18–30 years (OR = 0.56; 95% CI, 0.35–0.90) and those with endometrioma compared to those without endometrioma (OR = 0.56; 95% CI, 0.33–0.95) were more likely to be classified as members of the low pain phenotype. Conversely, individuals with low annual family income (≤ $19 999 vs. ≥ $100 000; OR = 2.00; 95% CI, 1.06–3.76), failure of previous treatment (vs. no failure; OR = 2.09; 95% CI, 1.29–3.39), painkiller use in the last 3 months (vs. no use; OR = 4.08; 95% CI, 2.40–6.93) and a first‐degree relative with a history of endometriosis or chronic pelvic pain (vs. none; OR = 1.94; 95% CI, 1.17–3.22) were more likely to be categorised in the high pain phenotype (Figure 2). A higher number of comorbidities was also predictive of membership in the high pain phenotype (age‐adjusted OR ‘aOR’: 1.40; 95% CI, 1.23–1.58), as well as the following comorbidities taken individually: painful bladder or interstitial cystitis (aOR = 2.25; 95% CI, 1.26–4.04), fibromyalgia (aOR = 4.11; 95% CI, 1.34–12.58), migraines (aOR = 1.83; 95% CI, 1.13–2.97), low back pain (aOR = 3.03; 95% CI, 1.55–5.93), irritable bowel syndrome (aOR = 3.60; 95% CI, 1.99–6.5), anxiety (aOR = 1.74; 95% CI, 1.07–2.84) and depression or mood disorders requiring medication or therapy (aOR = 1.81; 95% CI, 1.09–3.00) (Figure 3).

### 3.5 Association Between Phenotypes and Quality of Life

The mean quality of life score was higher in the high pain phenotype (59; 95% CI, 56–62) than in the low pain phenotype (33; 95% CI, 29–37), corresponding to a poorer quality of life. The overall Wald test indicated that phenotype membership was associated with distinct quality of life profiles (*p* < 0.001).

## 4 Discussion

### 4.1 Main Findings

This study identified two phenotypes in patients with endometriosis. One phenotype exhibited more severe and frequent pain with a poorer quality of life, whereas the other experienced milder and less frequent pain. These phenotypes demonstrated distinct clinical characteristics and predictors.

### 4.2 Interpretation and Implications for Clinical Practice and Research

Our findings are in line with studies highlighting the importance of using a broad set of biopsychological indicators to identify distinct subgroups of patients. Indeed, a growing body of research supports the hypothesis of the existence of hidden subgroups in patients with pain syndromes [18, 19, 39, 40, 41, 42, 43, 44, 45] or in female patients with chronic pelvic pain [46, 47, 48]. Previous studies have revealed the existence of two [18, 43, 48, 49] to nine [45] subgroups in patients with chronic pain, with no studies specifically examining the endometriosis population.

Current classification systems for endometriosis (revised American Society for Reproductive Medicine [6], ENZIAN [7] and American Association of Gynecologic Laparoscopists [8] classifications) are poorly correlated with the severity of pain symptoms [10]. Distinct clinical characteristics and predictors may facilitate the identification of individuals who manifest a more severe form of the disease and could potentially benefit from more comprehensive and specialised care.

The use of painkillers, as well as previous treatment failures, was predictive of high pain phenotype membership. This group may exhibit increased resistance or intolerance to first‐line therapy for endometriosis and may demonstrate a greater propensity for more complex manifestations of pain. It should be emphasised that a subset of patients with endometriosis may develop a more complex and prolonged pain condition despite comprehensive treatment, which is potentially indicative of central sensitization or nociplastic pain mechanisms [12, 50]. This population could benefit from consultation with an endometriosis specialist to explore second‐line therapies and complementary approaches to pain. A first‐degree relative with endometriosis or pelvic pain and the presence of comorbidities are predictors of the high pain phenotype. Additionally, a higher number of comorbidities was associated with this phenotype. These findings highlight the need for specialised care in this population, possibly requiring a multidisciplinary team [51]. Our findings are alarming, as young individuals aged 18–30 years, typically in a vital phase of their lives, are more likely to experience severe pain symptoms than women aged 30–41 years.

The presence of endometrioma predicted low pain phenotype membership, highlighting a recent discovery in endometriosis research. The largest systems biological study on endometriosis genetics found that ovarian endometriosis is genetically distinct from other types of endometriosis [52]. Our study assessed any endometriosis subtype, regardless of other lesions, and evaluated the presence of endometriomas with or without deep or superficial lesions. It is crucial but challenging to select and study appropriate cases for the three pelvic endometriosis subtypes. Endometrioma often co‐occurs with deep endometriosis, especially with severe pain [53], but not always [54]. We found insufficient evidence to determine whether individuals with superficial or deep endometriosis were more or less likely to belong to either pain phenotype.

Phenotypes can inform the development of more effective personalised interventions [55, 56]. These phenotypes may represent unobserved pain ‘typologies’ tied to different potential but unconfirmed pathogenic pathways or specific molecular and biochemical mechanisms. Phenotypes were linked to the outcome (quality of life). While the EHP‐30 questionnaire empowers patients to express the impact of the disease on their well‐being [57], our findings could help healthcare professionals make informed decisions and manage the disease clinically. Utilising the EHP‐30 questionnaire and pain phenotypes could improve the monitoring of progression, resolution and recurrence in patients with endometriosis [39, 57]. Moreover, clinical pain classification sets the extent of a condition in a way that is transportable from one centre to another [39, 58].

### 4.3 Strengths and Limitations

The strengths of this study include the focus on a specific population of patients with endometriosis, the use of validated tools [57] to provide input variables and outcomes, and the use of an innovative statistical method to analyse the data. Highlighting that sample size is an evolving area of study in the latent class analysis literature, the size of our sample was acceptable, as it is somewhat consistent with the literature. Based on numerous studies, it has been suggested that 300 or more cases are desirable; however, smaller samples may be adequate with simpler models (fewer input variables and classes) and ‘well‐separate’ classes [35, 36]. Although our sample size was adequate [35, 36], we acknowledge that initiatives to facilitate multicentre national and international collaborations using validated questionnaires could help identify a larger variety of phenotypes when incorporating additional indicators, such as sociocultural factors and biomarkers.

Some limitations of this study should be considered when interpreting our findings. At the assignment level, the mathematical algorithm provides only probability values for any patient to be in any of the potential phenotypes, and the phenotype membership is assigned based on the highest probability. That is, the participants do not belong to a single group. However, it should be emphasised that the average probabilities of an individual being assigned to a specific class, given their scores on the indicator variables, were more than acceptable (i.e., > 0.9) [35]. Comorbidity assessment may introduce detection bias, particularly for people who have had more contact with the medical system. Comorbidities, treatment failure, use of hormonal treatment or painkillers, quality of life and pain intensity were self‐reported and were related to some delay between each event and the survey. Memory limitations and selective recall may have affected some of the participants. This may have introduced a recall bias and misclassification. However, short recall periods (less than 3 months) are likely to have reduced reliance on participants' retrospective recall. Cross‐validation of the selected two‐phenotype solution is still required [35]. Furthermore, the reproducibility of this method should be tested using different source data [35]. It is important to note that applying latent class analysis to a cohort with a greater number of observed input variables or different input variables may result in the identification of a different number of classes with different patterns. Therefore, the phenotypes identified in our study were exclusively associated with the input variables used to infer them. Various case definitions of endometriosis were considered for inclusion in the study, including diagnoses based on symptoms and physical examination or visualisation using imaging techniques. This could result in misdiagnosed or undiagnosed cases of endometriosis (e.g., participants who were asymptomatic or diagnosed incidentally during unrelated pelvic surgical procedures, such as appendectomy or tubal ligation), thus introducing a potential for selection bias. This study is limited by its cross‐sectional design, which prevents the ability to establish causal relationships and assess phenotype stability or transition over time. Latent transition analysis represents a valuable methodology for examining the way individuals transition between latent classes (or phenotypes) of endometriosis. These data can provide crucial insights into the progression, stability and potential for improvement or exacerbation of symptoms associated with the disease. It must be acknowledged that the participants in this study were selected from a particular clinical setting and geographical area. Therefore, the findings may not be wholly representative of the experiences of women with endometriosis more widely or of women with this condition from diverse cultural backgrounds around the globe.

## 5 Conclusion

Patients with endometriosis can be categorised into two phenotypes that are distinctly associated with patient characteristics and quality of life. These findings require validation in other patient populations and may improve the understanding and identification of endometriosis variations, informing the development of more targeted interventions.

## Author Contributions

Fleur Serge Kanti and Sarah Maheux‐Lacroix contributed to study conception and design. Data analysis was performed by Fleur Serge Kanti. Data interpretation was performed by all authors. The manuscript was drafted by Fleur Serge Kanti and revised critically by all authors. The final approval of the version to be published was provided by all authors.

## Ethics Statement

Ethical approval (reference number: 2020‐4972) was obtained from the Research Ethics Committee of the *Centre Hospitalier Universitaire de Québec‐Université Laval*. Written informed consent was obtained from all the participants.

## Conflicts of Interest

The authors declare no conflicts of interest.

## Supporting information


Data S1.

## Data Availability

The data that support the findings of this study are available on request from the corresponding author. The data are not publicly available due to privacy or ethical restrictions.
