# Supplementary material for: Pain Phenotypes in Endometriosis: A Population‐Based Study Using Latent Class Analysis
Source: BJOG. 2024 Dec 3;132(4):492–503. doi: 10.1111/1471-0528.18021 (PMC11794060; doi:10.1111/1471-0528.18021)
Supplement: Supplementary file 1 — Data S1. [file BJO-132-492-s001.pdf]

SUPPLEMENTARY MATERIALS

Pain phenotypes in endometriosis and their associations with demographics, clinical characteristics, comorbidities and pain-related quality of life: a population-based study using latent class analysis

Authors and affiliation

Fleur Serge Kanti[1]
Valérie Allard[1]
Andrée-Ann Métivier[1]
Kristina Arendas[1]
Madeleine Lemyre[1]
Sarah Maheux-Lacroix[1]

1. Centre hospitalier universitaire de Québec - Université Laval, Quebec City, Quebec, Canada

Corresponding author

Sarah Maheux-Lacroix
Centre de recherche du CHU de Québec - Université Laval
2705, boulevard Laurier, Québec, Québec, Canada, GIV 4G2
+1 418 525 4444
sarah.maheux-lacroix@crchudequebec.ulaval.ca

Contents

Methods S1 3

Latent class analysis 3

Covariates and outcome association analyses 4

Table S1. Class enumeration 7

Table S2. Average latent class probabilities for most likely latent class membership (row) by latent class (column) 8

Table S3. Descriptive statistics of intensity of symptoms of pain used as input in the latent class analysis stratified by the identified pain phenotypes. 9

Figure S1. Plot of conditional item probabilities 10

Supplementary references 11

Methods S1

Latent class analysis

To identify phenotypes of pain, latent class analysis (LCA) was conducted based on the indicator variables described above. LCA is a data-driven statistical method used to uncover unobserved heterogeneity in a population and identify homogeneous subgroups or latent classes of people (i.e., phenotypes) that are similar in their responses to a chosen set of measured indicators. LCA was selected for its "person-centered" approach. Probabilities of class membership are obtained, and individuals are assigned to classes based on their probability of being in classes given the pattern of scores they have on indicator variables. In contrast, "variable-centered" approaches (e.g., factor analysis) look for relationships among variables, and individuals are represented along dimensions. Moreover, compared to other classification techniques such as cluster analysis or k-means clustering, LCA is model-based and enables a mathematical assessment of how a proposed LCA model accurately represents/fits the data [1–3]. We took the one-class model as a baseline, increasing the number of classes until the optimal class solution model was reached [3,4]. Model fit statistics guided the selection of the optimal fitting model. The Bayesian Information Criteria (BIC) and the Bootstrap Likelihood Ratio Test (BLRT) were prioritized [1,4]. The following fit indexes and tests were reported: the Akaike Information Criterion (AIC), the adjusted BIC (aBIC), Lo–Mendell–Rubin Likelihood Ratio Test (LMRLRT), and entropy. The lower values of the BIC, AIC, and aBIC indicate a better fitting model. A non-significant value of LMRLRT and BLRT, obtained after comparing a k-class model with a k–1-class model, indicates that a more parsimonious model should be kept. Entropy informs about the classification accuracy, values closer to one indicating clear delineation of classes [3–5]. Individuals were then assigned to a specific class based on their highest estimated posterior probability of class membership (this is the most

likely latent class membership). This meant that an individual with hypothetical posterior probabilities of 5%, 10%, 40%, 30 and 15% of belonging to classes 1, 2, 3, 4 and 5, respectively, was assigned to class 3. The final counts and proportions of individuals in each class were calculated. Conditional item probabilities (proportions endorsing each category of each of all indicator variables conditional on class membership) were plotted. To aid interpretation, descriptive labels were assigned to the identified classes. To do so, we visually inspected the item probability plot and examined the qualitative differences among the classes. The full-information maximum likelihood approach was used to handle missing data [5]. This uses the information available for each participant to maximize the sample log-likelihood function for estimating parameters and standard errors, under the assumption that the indicators are missing at random [6].

Covariates and outcome association analyses

Post hoc descriptive analyses of demographics, clinical characteristics and comorbidities across classes were performed to produce a demographic, clinical, and comorbidity profile for each class. The estimates were based on the most likely latent class membership of participants. This consideration does not consider uncertainty around the estimated posterior probabilities of latent class assignment for each participant. Post hoc statistical comparisons for these variables across classes would have high type I error rates and were not appropriate. Therefore, we did not perform these comparisons. The participants' (demographic and clinical) characteristics and comorbidities were tested as potential predictors of class membership (phenotype predictors) using logistic regression models in a bias-adjusted three-step procedure. This approach allows to directly examine the predictors without imposing bias to the class solution [7–9]. Unadjusted (for all predictors) and age-adjusted (for comorbidities) odds ratios were estimated.

The three-step method was also considered for the outcome variable (as a LCA distal variable). The automatic approach was used to estimate class-specific means of quality of life for each of the two classes identified in LCA and with the produced overall Wald test to examine whether classes or phenotypes of pain were associated with quality of life. The estimates were supplemented by 95% confidence intervals. Significant differences were assessed by examining the confidence intervals (an odds ratio is significantly different from 1 if its confidence interval does not include 1).

Briefly, the three-step method for predictors of the latent class variable is as follows [16,18].

1. First, a regular LCA using only the latent class indicators/inputs was performed (LCA without covariates/predictor variables). This is the measurement model.
2. Second step: Using the latent class posterior distribution obtained during the LCA estimation (first step), an external variable accounting for classification uncertainty (nominal variable *N*) was created to represent the most likely class variable. For each observation, *N* is set to class *c*, for which the probability $P(C = c|U)$ is the largest, where *U* represents the latent class indicators, and *C* is the latent class variable. Briefly, the second step produces *N*, an imperfect measurement of *C* with a measurement error *e*.
3. Third step: An auxiliary variable *X* (e.g., demographics, clinical characteristics, and comorbidities) is included, and the most likely class variable *N* is used as the latent class indicator variable *C* with uncertainty rates prefixed at the measurement error *e*. The measurement relationships between the latent class variable *C* and the nominal most likely class variable *N* are fixed, while the parameters of the multinomial regression of *C* on predictor *X* are estimated. This step uses a multinomial logistic regression in instances where there are three or more phenotypes, or a logistic regression model in cases where there are two phenotypes.

The method is extended to distal outcomes Y (e.g., quality of life), which are predicted by the latent class variable.

Table S1. Class enumeration

k classes	Loglikelihood	BIC	BLRT	VLRT	AIC	aBIC	Entropy	Size per class
1	-2739.762	5567.478	-	-	5509.523	5519.892	-	352
2	-2502.305	**5186.383**	<0.0001	<0.0001	5066.610	5088.039	0.820	189/163
3	-2470.292	5216.175	<0.0001	0.07	5034.584	5067.073	0.788	181/90/81
4	-2437.300	5244.008	<0.0001	0.08	5000.599	5044.147	0.785	136/101/68/47
5	-2415.662	5294.552	0.04	0.33	4989.325	**5043.933**	0.822	134/91/68/35/24
6	-2395.892	5348.829	0.2	0.2	4981.784	5047.452	0.825	130/79/65/36/26/16
7	-2376.371	5403.606	0.03	0.46	**4974.743**	5051.471	0.838	81/81/57/57/34/26/16
8	-2360.954	5466.589	0.07	0.76	4975.908	5063.696	0.849	74/57/53/46/40/38/27/17
9	-2346.629	5531.758	0.33	0.76	4979.258	5078.106	0.858	77/75/47/35/31/27/23/21/16
10	-2331.240	5594.797	<0.0001	0.76	4980.480	5090.388	0.881	74/45/41/40/37/37/28/22/20/8

Abbreviations: k, number of classes; BIC, Bayesian Information Criterion; BLRT, parametric bootstrapped likelihood ratio test for k-1 classes (H_0) versus k classes (H_1); VLRT, Vuong-Lo-Mendell-Rubin likelihood ratio test for k-1 classes (H_0) versus k classes (H_1); AIC, Akaike Information Criterion; aBIC, adjusted Bayesian Information Criterion.

Notes: Bolded values of BIC, AIC and aBIC indicate "best" fit for each respective statistic. P-values of BLRT or VLRT < 0.05 indicate the goodness of fit of the k-class model relative to the k-1-class model. The 2-class model was selected as final model based on low BIC and p-value of BLRT < 0.05 for the goodness of fit of the 2-class model relative to the 1-class model.

Table S2. Average latent class probabilities for most likely latent class membership (row) by latent class (column)

Most likely latent class membership	Latent class	
	1	2
1	0.953	0.047
2	0.059	0.941

The average latent class posterior probability represents the mean likelihood that the class model accurately predicts group membership for individuals. The average latent posterior probabilities are presented in a matrix, with the diagonal values indicating the average probability of an individual being assigned to a specific class, given their scores on the indicator variables used to define the classes [11]. Higher diagonal probabilities (i.e., closer to 1.0) are preferred. The off-diagonal elements in the posterior probability matrix reflect the likelihood of cases belonging to one class being assigned to another class within the current solution. Lower off-diagonal values (i.e., closer to 0) are desirable. Some researchers employing latent class analysis utilize a cutoff of .80 for acceptable diagonal probabilities [12], while others suggest a threshold greater than .90 [11]. The researchers agree that a value exceeding .90 is ideal; however, if other criteria are met and the model is theoretically supported, probabilities between .80 and .90 may be considered acceptable. Although meeting the .90 criterion for all average latent class posterior probabilities is not essential, provided that other requirements are fulfilled, values below .80 should be viewed as unacceptable [3].

Table S3. Descriptive statistics of intensity of symptoms of pain used as input in the latent class analysis stratified by the identified pain phenotypes.

Pain intensity	Pain phenotypes		Total (N=352)
	Low (class 1) n=163(46.3%)	High (class 2) n=189(53.7%)	
Superficial dyspareunia			
Mean ± SD	1.9±2.7	3.2±3.3	2.6±3.1
Median (minimum - maximum)	0(0-10)	2(0-10)	1(0-10)
25%-75% percentiles	0-3	0-6	0-5
Deep dyspareunia			
Mean ± SD	4±3.2	6.5±2.7	5.3±3.2
Median (minimum - maximum)	4(0-10)	7(0-10)	6(0-10)
25%-75% percentiles	0-7	5-8	3-8
Dysmenorrhea			
Mean ± SD	3.8±3.3	6.7±3.5	5.4±3.7
Median (minimum - maximum)	3(0-10)	8(0-10)	6(0-10)
25%-75% percentiles	0-7	6-9	1-9
Dyschezia			
Mean ± SD	2.4±2.7	4.6±2.9	3.5±3
Median (minimum - maximum)	1(0-10)	5(0-10)	3(0-10)
25%-75% percentiles	0-5	2-7	0-6
Acyclic pelvic pain			
Mean ± SD	3.6±3.1	6.5±2.7	5.2±3.2
Median (minimum - maximum)	4(0-10)	7(0-10)	6(0-10)
25%-75% percentiles	0-6	5-8	3-8

Abbreviations: SD, standard deviation

Pain intensity is the numerical rating scale score ranging from 0 (indicating no pain) to 10 (indicating the worst pain imaginable)

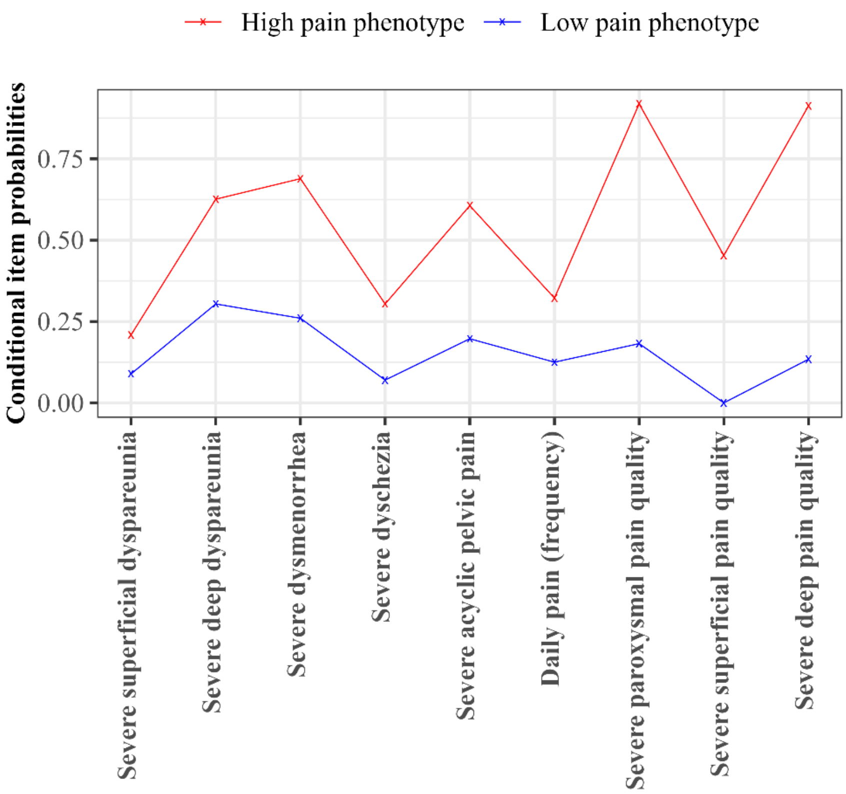

Figure S1. Plot of conditional item probabilities

Conditional item probabilities were proportions endorsing each category of the indicator variables conditional on class membership. The category indicators plotted were the daily frequency of pelvic pain, severe intensity of superficial dyspareunia, deep dyspareunia, dysmenorrhea, dyschezia, and acyclic pelvic pain in the past three months, and severe intensity of paroxysmal, superficial, and deep pain quality.

Supplementary references

1. Nylund-Gibson K, Choi AY. Ten frequently asked questions about latent class analysis. Translational Issues in Psychological Science. 2018;4(4):440–61.

2. Miettunen J, Nordström T, Kaakinen M, Ahmed AO. Latent variable mixture modeling in psychiatric research – a review and application. Psychological Medicine [Internet]. 2016 Feb [cited 2024 Apr 24];46(3):457–67. Available from: https://www.cambridge.org/core/journals/psychological-medicine/article/latent-variable-mixture-modeling-in-psychiatric-research-a-review-and-application/2608C356F80590CB99C06CCAA401D8B8

3. Weller BE, Bowen NK, Faubert SJ. Latent Class Analysis: A Guide to Best Practice. Journal of Black Psychology [Internet]. 2020 May 1 [cited 2024 Apr 20];46(4):287–311. Available from: https://doi.org/10.1177/0095798420930932

4. Nylund KL, Asparouhov T, Muthén BO. Deciding on the Number of Classes in Latent Class Analysis and Growth Mixture Modeling: A Monte Carlo Simulation Study. Structural Equation Modeling: A Multidisciplinary Journal [Internet]. 2007 Oct 23 [cited 2024 Apr 24];14(4):535–69. Available from: https://doi.org/10.1080/10705510701575396

5. Sinha P, Calfee CS, Delucchi KL. Practitioner's Guide to Latent Class Analysis: Methodological Considerations and Common Pitfalls. Critical Care Medicine [Internet]. 2021 Jan [cited 2024 Apr 24];49(1):e63. Available from: https://journals.lww.com/ccmjournal/abstract/2021/01000/practitioner_s_guide_to_latent_class_analysis_.26.aspx

6. Thoemmes F, Rose N. A Cautious Note on Auxiliary Variables That Can Increase Bias in Missing Data Problems. Multivariate Behavioral Research [Internet]. 2014 Sep 3 [cited 2024 Apr 24];49(5):443–59. Available from: https://doi.org/10.1080/00273171.2014.931799

7. Muthén LK, Muthén BO. Mplus User's Guide [Internet]. 8th ed. Los Angeles, CA: Muthén & Muthén; 1998 [cited 2024 Apr 25]. 950 p. Available from: https://www.statmodel.com/download/usersguide/MplusUserGuideVer_8.pdf

8. Asparouhov T, Muthén B. Auxiliary Variables in Mixture Modeling: Three-Step Approaches Using Mplus. Structural Equation Modeling: A Multidisciplinary Journal [Internet]. 2014 Jul 3 [cited 2024 Apr 24];21(3):329–41. Available from: https://doi.org/10.1080/10705511.2014.915181

9. Bakk Z, Vermunt JK. Robustness of Stepwise Latent Class Modeling With Continuous Distal Outcomes. Structural Equation Modeling: A Multidisciplinary Journal [Internet]. 2016 Jan 2 [cited 2024 Apr 24];23(1):20–31. Available from: https://doi.org/10.1080/10705511.2014.955104

10. Asparouhov T, Muthen B. Auxiliary Variables in Mixture Modeling: Using the BCH Method in Mplus to Estimate a Distal Outcome Model and an Arbitrary Secondary Model [Internet]. 2021. Available from: https://www.statmodel.com/examples/webnotes/webnote21.pdf

11. Muthén B, Muthén LK. Integrating Person-Centered and Variable-Centered Analyses: Growth Mixture Modeling With Latent Trajectory Classes. Alcoholism: Clinical and Experimental Research [Internet]. 2000 [cited 2024 Jul 10];24(6):882–91. Available from: https://onlinelibrary-wiley-com.acces.bibl.ulaval.ca/doi/abs/10.1111/j.1530-0277.2000.tb02070.x

12. Weden MM, Zabin LS. Gender and Ethnic Differences in the Co-occurrence of Adolescent Risk Behaviors. Ethnicity & Health [Internet]. 2005 Aug 1 [cited 2024 Jul 10];10(3):213–34. Available from: https://doi.org/10.1080/13557850500115744
